# Risk assessment of failure during transitioning from in-centre to home haemodialysis

**DOI:** 10.1186/s12882-022-03039-4

**Published:** 2022-12-20

**Authors:** Sabrina-Wong-Peixin Haroon, Titus-Wai-Leong Lau, Gan Liang Tan, Eugene-Hern Choon Liu, Soh Heng Hui, Siao Luan Lim, Diana Santos, Robyn Hodgson, Lindsay Taylor, Jia Neng Tan, FH HHD, Andrew Davenport

**Affiliations:** 1 Division of Nephrology, National University Hospital Singapore, Level 10, NUHS Tower Block, 1E Kent Ridge Road, Singapore, 119228 Republic of Singapore; 2 Department of General Medicine, Sengkang General Hospital, Singapore, Singapore; 3 Department of Anaesthesia, Yong Loo Lin School of Medicine, National University of Singapore, Singapore, Singapore; 4 Renal Centre, National University Hospital, Singapore, Singapore; 5 Medical Affairs-Clinical Governance, National University Hospital Singapore, Singapore, Singapore; 6 Department of Renal Medicine, University College London, Royal Free Hospital, London, United Kingdom

**Keywords:** Home haemodialysis, Safety, Vascular access, Failure mode and effect analysis, Adverse events

## Abstract

**Background:**

Introducing a de-novo home haemodialysis (HHD) program often raises safety concerns as errors could potentially lead to serious adverse events. Despite the complexity of performing haemodialysis at home without the supervision of healthcare staff, HHD has a good safety record. We aim to pre-emptively identify and reduce the risks to our new HHD program by risk assessment and using failure mode and effects analysis (FMEA) to identify potential defects in the design and planning of HHD.

**Methods:**

We performed a general risk assessment of failure during transitioning from in-centre to HHD with a failure mode and effects analysis focused on the highest areas of failure. We collaborated with key team members from a well-established HHD program and one HHD patient. Risk assessment was conducted separately and then through video conference meetings for joint deliberation. We listed all key processes, sub-processes, step and then identified failure mode by scoring based on risk priority numbers. Solutions were then designed to eliminate and mitigate risk.

**Results:**

Transitioning to HHD was found to have the highest risk of failure with 3 main processes and 34 steps. We identified a total of 59 areas with potential failures. The median and mean risk priority number (RPN) scores from failure mode effect analysis were 5 and 38, with the highest RPN related to vascular access at 256. As many failure modes with high RPN scores were related to vascular access, we focussed on FMEA by identifying the risk mitigation strategies and possible solutions in all 9 areas in access-related medical emergencies in a bundled- approach. We discussed, the risk reduction areas of setting up HHD and how to address incidents that occurred and those not preventable.

**Conclusions:**

We developed a safety framework for a de-novo HHD program by performing FMEA in high-risk areas. The involvement of two teams with different clinical experience for HHD allowed us to successfully pre-emptively identify risks and develop solutions.

**Supplementary Information:**

The online version contains supplementary material available at 10.1186/s12882-022-03039-4.

## Background

The past decade has seen a growing literature reporting superior outcomes for home haemodialysis (HHD) patients compared to patients dialysing in-centre (ICHD) [1, 2]. The benefits of HHD extend beyond flexibility in scheduling, reduced travel time, and improved quality of life when receiving dialysis treatment in a familiar home environment [3, 4]. Recently published studies of HHD have even reported comparable survival to deceased donor renal transplant patients [5, 6]. Many renal programmes are now advocating HHD, in addition to peritoneal dialysis (PD), as the preferred modality choice for chronic maintenance dialysis patients.

Although HHD is not a new modality and has a good safety record in established programmes [7, 8], Singapore is only just initiating a pilot programme. Our programme will offer suitable local end stage kidney failure (ESKF) patients who are already receiving haemodialysis (HD) or have chosen HD as their long-term option to be treated at home. The adoption of home dialysis has been slow, in part because Singapore is geographically small and well connected with one of the world’s leading public transportation systems. Older patients have also been reluctant to accept that dialysis treatments can be performed safely at home. Fortunately, with wider exposure and better health literacy over recent years, there are now an increasing number of patients keen to participate in HHD as a long-term dialysis modality [9–11].

The fundamentals of HD are essentially the same, whether ICHD or HHD. In Singapore 80% of patients with chronic kidney disease treated by dialysis attend ICHD [12]. For our pilot trial of HHD, we excluded patients using tunnelled catheters for vascular access, given the higher risk of adverse incidents [13]. We decided to make a caregiver a requirement for the initial patients starting HHD to improve patient confidence while starting this new modality option to patients used to ICHD. The prerequisite requirement of a mandatory caregiver is more restrictive in allowing access to HHD. Although previously published reports do not demonstrate that the presence of a care giver translates into reducing the risk of adverse events [13] but a caregiver was chosen to allay the anxieties of patients and stakeholders.

For HHD, the major goals are to prepare the staff both as educators and trainers, the patient and the home ready for dialysis. As we are starting and adopting a new modality that will change the landscape of chronic maintenance dialysis locally in Singapore, we strengthened our HHD risk assessment by evaluating the risk of failure in the processes of HHD using the Failure Mode Effects Analysis (FMEA) tool. FMEA has been used in many healthcare settings, including ICHD, to assess various new critical policies and procedures before implementation and also to identify areas for improvement [14–17]. Our study will be the first to use FMEA to evaluate the risk of failure in HHD. We collaborated with one of the pioneers of HHD, the Royal Free Hospital in London, to further strengthen and validate our results. We aimed to review potential failures in the HHD process and identify the highest risk areas to help us develop prioritised interventions designed to prevent failures or at least minimise risks. We also defined remedial actions to mitigate the impact and consequences of any failures.

## Methods

We conducted a FMEA between July 2020 to February 2021 with teams from the National University Hospital Singapore (NUH) and Royal Free Hospital (RFH) in London. The FMEA was performed independently and adjusted collaboratively. We performed our FMEA in the following manner:

- Step 1: Selection of team members.
- Step 2: Process scope identification and listing of all key processes (using process flowchart).
- Step 3: Identification of failure mode.
- Step 4: Scoring based on risk priority numbers.
- Step 5: Designing solutions to eliminate and mitigate risk.

Ethics approval for the study was not required by The National Healthcare Group Institutional Review Board in Singapore as failure mode effect analysis does not meet the definition of human-subject research. All methods were performed in accordance with the relevant institutional guidelines and regulations.

### Step 1: team selection

We identified the key members for the FMEA process and recruited cross-functional members with diverse and in-depth knowledge of HD and HHD from both NUH and RFH. Teams from these two hospitals shared common expertise in HD but were distinct in that one is planning for the initiation of a HHD programme, whereas the other has decades of experience in HHD. Together, we provided unique perspectives of a HHD programme at different phases of maturity.

### Step 2: process identification and process flowchart preparation

We reviewed the established processes involved in the HHD journey at RFH and conducted a search on the HHD processes in PubMed. Each team identified and listed all key processes and subprocesses in HHD independently, and this was subsequently collated to map the final processes and subprocesses.

### Step 3: failure mode identification

We reviewed the HHD experience at the RFH and used “brainstorming” sessions and an extensive review of published literature to identify all the possible failure modes in HHD. We listed all potential failures that can occur at each step in the flowchart and found that the area with the highest risk involves the transition phase of patients from in-centre haemodialysis to home.

A list of potential failure modes during the process of transitioning to home was generated. The list was categorised, reviewed for accuracy and completeness by both the NUH and RFH teams. The possible areas of failure were identified, considering contributing factors and potential consequences.

### Step 4: scoring based on risk priority numbers

In the areas of greatest concern, we rated each process failure for likelihood (remote to very high), severity (none to catastrophic), and detectability (almost certain to absolutely uncertain) using a consensus approach. The risk priority number (RPN) was then calculated for each potential failure mode. The RPN is the quantitative estimate of the risk associated with each failure mode (Table 1) [18]. FMEA teams assigned an RPN to each failure mode based on three factors: (1) the likelihood of occurrence (L), (2) the degree of severity if it does occur (S), and (3) the likelihood of detecting the occurrence (D). The RPN was calculated using the formula: L x S x D, where high numbers indicate a high priority for intervention and action [19, 20]. The scores were determined based on consensus, following discussions between NUH and RFH team members. A failure mode with an RPN of 100 or greater was considered a high priority and was further investigated and documented in the FMEA worksheet (Table 2) [21, 22].

**Table 1:** Risk priority definition and rating scales [18]

Risk priority number	Definition	Rating scales
Likelihood	The perceived chance of the failure happening within a defined period	Rating of 1–10: ‘failure is unlikely’ to ‘very likely or inevitable’
Severity	How severe the outcome is to the patient should failure occur	Rating of 1–10: ‘no severity at all’ (would not affect individual or system) to ‘moderate’ (significant effect with no injury) to ‘major injury’ to ‘death’
Detectability	Is the area of failure readily known, or is it discovered only when an adverse outcome occurs?	Rating of 1–10: ‘almost certain the process or steps will detect potential cause(s)’ to ‘absolute uncertainty that the control will not detect potential cause(s) and subsequent failure mode (s)

**Table 2:** Possible areas of failure during transitioning to home haemodialysis

Steps	Failure mode
**1. Setting up HHD system at home**	
Setting up HD Machine	Machine cannot be placed in the space at home
Setting up water treatment system	Portable RO cannot be placed in the space at home
	Connection of portable RO and machine cannot be achieved
Setting up drainage	Violating municipal standards for discharge of dialysis effluent
	Incorrect technical requirement for height of drainage hole
Setting up and establishing power and water supply	Power socket not suitable for machine / portable RO
	Power socket not sited correctly
	Connector to water point incompatible
	Water points not sited correctly
	Inadequate water pressure to operate the portable RO
	Water temperature too high or too low
Creating storage and getting consumables ready	Lack of storage for consumables
	Consumables passed shelf-life (beyond expiration date)
**2. Completing test prior to starting HHD**	
Checking water treatment system	Failure to achieve meet minimum safety and quality levels of dialysis water and fluid requirement
Initiating HD machine self-test	Repeatedly fails self-test
**3. Performing HHD**	
**3a. Preparing HHD**	
Starting HD Machine	Machine cannot be switched on
	Machine breakdown
Starting water treatment system	Portable reverse osmosis (RO) cannot be switched on
	Portable RO breakdown
	Incorrect portable RO connection
Ensuring drainage	Inadequate water or dialysate flow
	Inadequate water pressure
	Blocked drainage
	Flooding from cracked lines or choked drainage
Starting power and water supply	Interruption in water supply
	Interruption in power supply
Gathering dialysis consumables	No or insufficient supply of consumables needed for treatment
	Incorrect supply of consumables
	Failure to supply heparin
	Failure to supply disinfectant
**3b. Evaluation before starting dialysis**	
General evaluation	Starting dialysis when feeling unwell or have temperature > 38^o^ C, heart rate > 110 or < 50 beats per minute, systolic blood pressure > 180 mmHg or < 100 mmHg
Priming and connectivity of dialysis blood lines	Poor connection
	Incorrect connection
	Kinked blood lines
Measuring weight	Error in weight taken
	Incorrect dry weight
Deciding and calculation of ultrafiltration	Excessive ultrafiltration
	Inadequate ultrafiltration
Taking medications before dialysis	Taking excessive antihypertensive medications
	Forgot to take antihypertensive or taking lower dose
**3c. Managing vascular access during dialysis treatment**	
Cleaning of access site	Non-compliance to cleaning of access site
Scab removal for those of buttonhole cannulation	Incomplete scab removal for buttonhole cannulation
Establishing access cannulation	Unsuccessful access cannulation after three attempts
Cannulation technique	Defective technique in cannulation access
Securing vascular access	Poor fixation of needles to skin, traction of circuit line or movement especially during nocturnal dialysis
Troubleshooting alarm related to vascular access	Failure to respond to arterial and venous pressure alarm
Monitoring vascular access during dialysis	Failure to monitor vascular access during dialysis
Vascular access needles removal	Excessive and prolonged bleeding after removal of dialysis needles
Monitoring vascular access (general)	Failure to identify access related infection
**3d. Interruption and management of machine alarms**	
Reprogramming after temporary interruption	Failure to reprogram after disconnection
Troubleshooting dialysis machine alarms	Dialysate (conductivity and temperature) alarm trigger
	Air detection alarm trigger
	Blood leak alarm trigger
Calling for help	Unable to reach nursing or technical assistant for advice
Emergency during dialysis treatment	Need for emergency evacuation
**3e. Administering medications on dialysis**	
Administering anticoagulation	Excessive heparin administered
Administering new medications or using new consumables	Allergic reaction
**3f. Other**	
Caregiver assisting HHD	Needle-stick injury to family member or caregiver
**3g. Ending dialysis**	
Disposal of HD items	Improper of disposal biohazard waste
	Sharps box missing

### Step 5: designing solutions to eliminate, mitigate risk and risk review

For the highest area of concern, potential solutions to mitigate risks and interventions were evaluated. As risk mitigation in this area usually involved a bundle approach and so was often overlapping, we discussed all possible solutions for each failure in the area concerned. We had extensive discussions for areas with RPN scores more than 100. A risk review process is planned prospectively as the HHD program starts to refine the FMEA.

## Results

The NUH team consisted of three nephrologists with one having formal training in patient safety and healthcare quality, a senior renal nurse manager, one newly trained HHD nurse, a representative from hospital clinical governance experienced in conducting regular FMEA, an administrative executive, and a non-renal physician trained in healthcare quality from another independent (non-affiliated) institution. The team from RFH consisted of a nephrologist, two nurse managers of the HHD and home therapies programs, two senior renal technologists, and two patients each with more than 10 years HHD experience. The teams had 5 meetings of approximately 2 h. FMEA was conducted separately by each team and then through video conferencing platform (ZOOM) meetings.

### Flowchart of key processes in HHD

**Fig. 1:**
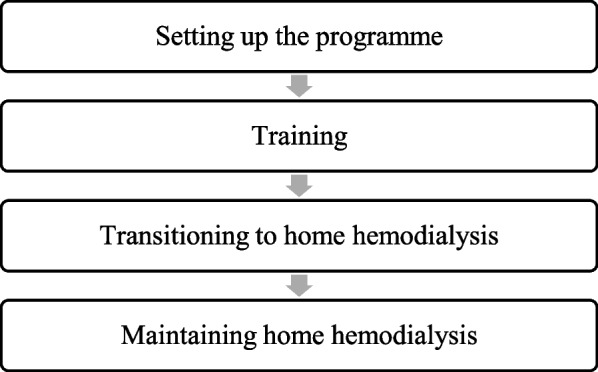
Flowchart of setting up home haemodialysis programme

We identified all the key areas, processes and sub-processes in setting up HHD. The four major areas in HHD involve setting up the program, training of staff and patients, transferring patients from in-centre to HHD, and ongoing maintenance therapy (Fig. 1).

### Failure modes and risk priority numbers

After reviewing various main areas, transitioning to HHD was found to have the highest risk of failure (Fig. 1). The FMEA on the transitioning to HHD process was independently completed by the two teams, but the final score was determined after joint deliberation. The three main processes in transitioning to HHD area are setting up the HHD system at home, completing tests before starting HHD, and performing HHD.

There was a total of 5 main steps with 13 failure modes in setting up the HHD system at home and 2 main steps with 2 failure modes in completion of the tests before starting HHD. We identified that performing HHD treatment has a total of 7 sub-processes; including (1) preparation to start HHD treatment, (2) general patient and dialysis equipment evaluation before starting dialysis, (3) managing vascular access during dialysis treatment, (4) dealing with treatment interruption and troubleshooting machine alarms, (5) administering medication during dialysis, (6) others, and (7) ending dialysis, with a total of 27 steps and 44 failure modes (Table 2). Among the sub-processes, managing vascular access during treatment has the highest failure modes (Additional file 1: Appendix 1).

Vascular access management had a total 9 steps with 9 failure modes. Failure mode effect analysis revealed scores ranging from 9 to 256 with a mean of 135 and median of 144 respectively. The highest RPNs were failure to monitor vascular access, a defective technique in cannulation access, and troubleshooting vascular access alarm with scores of 252, 256, and 180 respectively. As vascular access management and troubleshooting access alarms are overlapping and have higher RPN scores, we explored the failure modes, effect, consequences, and identified risk mitigation strategies in all 9 areas of access-related medical emergencies. In particular we emphasized 3 main areas with high RPN scores, highlighted in grey in Table 3.

**Table 3:** Failure modes of access related medical emergencies

Number	Steps	Failure mode	Effect/ Consequences	Likelihood	Severity	Detectability	RPN
1	Cleaning of access site	Non-compliance to cleaning of access site	Vascular access infection and septicaemia Unable to proceed with haemodialysis using vascular access	2	6	6	72
	Potential solutions for risk mitigation 1. Education and training to patient with emphasis on infection prevention measures [23, 24] 2. Performing hand hygiene before setting up table to start HHD 3. Emphasize hand washing and use of chlorohexidine swab sticks or consumables that motivate compliance 4. Regular assessment of patient performing treatment observed during home visit and during physician/home haemodialysis nursing team review at HHD unit (at least once every two months)						
2	Scab removal for those of buttonhole cannulation	Incomplete scab removal	Vascular access infection and septicaemia Unable to proceed with haemodialysis using vascular access	2	9	8	144
	Potential solutions for risk mitigation 1. Preferential selection for rope ladder cannulation [25] 2. Careful consideration on suitability of buttonhole cannulation only in selected cases and strictly avoiding individuals that are recurrent methicillin-sensitive staphylococcus aureus (MSSA) methicillin-resistant staphylococcus aureus (MRSA) colonised 3. Conversion to rope ladder cannulation method for those using button-hole cannulation if MSSA or MRSA colonised 4. Specific training for buttonhole cannulation with emphasis on infection prevention measures [26] 5. Monitoring MRSA colonisation status and eradication (when needed) every three months [27–30] 6. Topical mupirocin ointment to buttonhole cannulation sites [31] 7. Regular assessment of patient performing treatment observed during home visit and during physician/ home haemodialysis nursing team review at HHD unit (at least once every two months)						
3	Establishing access cannulation	Unsuccessful cannulation after 3 attempts	Unable to proceed with haemodialysis	3	3	1	9
	Potential solutions for risk mitigation 1. Education and training with individualised cannulation plan 2.Contact HHD hotline and when necessary, report to training center 3. Re-training for cannulation technique if needed						
4	Cannulation technique	Defective technique in cannulating access	Acute blood loss from venous extravasation and hematoma Vascular access infection and septicaemia Unable to proceed with haemodialysis using vascular access	4	9	7	252
	Potential solutions for risk mitigation 1. Dedicated staff for training 2. Individualised cannulation technique and type 3. Competency check during training and maintenance phase using audit tool						
5	Securing vascular access	Poor fixation of needles to skin, traction of circuit line or movement especially with nocturnal dialysis	Anaemia symptoms Acute blood loss	2	10	5	100
	Potential solutions for risk mitigation 1. Discussion with patient regarding dialysis treatment plan (day time or nocturnal) and customized securing vascular access technique 2. Adequate cleaning and drying of area before cannulation [32] 3. Butterfly style taping method preferentially or in selected patient Chevron styles [33, 34] 4. Blood lines looped loosely to allow movement of patient and prevent blood lines pulling on needles especially in nocturnal dialysis [32] 5. Setting the lower limit of the venous pressure alarm as close as possible to current venous pressure alarm 6. Wetness detector, especially for nocturnal haemodialysis 7. Regular assessment of patient performing treatment observed during home visit and during physician review at HHD unit (at least once every two months)						
6	Troubleshooting alarm related to vascular access	Failure to respond to arterial and venous pressure alarm	Acute blood loss Hypotensive shock and death if excessive blood loss	3	10	6	180
	Potential solutions for risk mitigation 1. Training to emphasise importance, consequences in addition to troubleshooting 2. Competency check during training and maintenance phase 3. Short concise patient education card 4. Additional devices to detect blood loss 5. A bell to notify caregiver to provide help and have a (mobile) telephone within reach to call for help						
7	Monitoring vascular access during dialysis	Failure to monitor vascular access during dialysis	Vascular access thrombosis Bleeding from needling sites Vascular access rupture Acute blood loss causing hypotensive shock and death if excessive blood loss Unable to proceed with haemodialysis using vascular access	4	8	8	256
	Potential solutions for risk mitigation 1. Training to patient to do basic monitoring and report on signs of infection with a clinical tool, such as Mr Victor (for dialysis catheters exit site), and pseudo-aneurysm development, with photographic evidence as appropriate 2. Emergency education to use an inverted bottle top and bandaging to limit hemorrhage from a ruptured fistula 3. Training nursing staff to perform physical examination during patient encounter (at least once every two months) 4. Appropriate pump speeds [26] 5. Vascular access monitoring during clinic review 6. Cannulation sites are determined by home HD staff along with vascular surgeon including identifying unsafe sites not for cannulation 7. Close collaboration with vascular access team 8. Call emergencies services and report to hospital emergency department if bleeding or access rupture occurs						
8	Vascular access needles removal	Excessive and prolonged bleeding after removal of dialysis needles (bleeding after direct pressure applied for 10 min) [35, 36]	Anaemia symptoms Acute blood loss	2	5	5	50
	Potential solutions for risk mitigation 1. Education and training to patient 2. Avoid excessive anticoagulation dosages [37] 3. Removal of the needles in sequences, one needle at a time once there is no bleeding 4. Training nursing staff to perform physical examination during patient encounter (initially at least once every two months) 5. Vascular access monitoring during clinic review to exclude proximal stenosis						
9	Monitoring dialysis access (general)	Failure to identify access related infection	Unable to proceed with haemodialysis using vascular access	2	8	5	80
	Potential solutions for risk mitigation 1. Education and training to patient 2. Training nursing staff to perform physical examination during patient encounter (initially at least once every two months) 3. Patient to check for access viability before attempting cannulation 4. Vascular access monitoring during clinic review						

### Risk mitigation strategies

Table 3 provides strategies for risk mitigation of failure modes in the 9 areas related to vascular access. We identified 46 risk mitigation strategies. We discussed all areas related to vascular access with focus on the three main steps with the highest RPN (grey shading in Table 3). Some of the potential strategies for risk mitigation were overlapping.

The most significant concern was a defective technique in access cannulation that usually occurs most frequently during the early transitioning phase to HHD. We suggest risk reduction in this area needs to be initiated at the start of HHD training. In addition to identifying trained staff dedicated to HHD training, we propose an individualised vascular access approach to self-cannulation for each patient [26, 38–40]. The individualised approach includes a single dedicated trainer, type of techniques, cannulation type, and weekly review during the first three months of HHD training by a designated HHD nurse with subsequent discussion with the HHD team at weekly meetings. The most suitable cannulation technique will help overcome the fear associated with cannulation and improve confidence in cannulation [41–43]. Rope-ladder cannulation remains the preferred cannulation technique for HHD, and our programme encourages this over buttonhole cannulation [44]. Although buttonhole cannulation is possible for HHD patients self-needling and patients with short segment for cannulation, it should be avoided in MSSA and MRSA colonised patients [45, 46]. Patients that are MSSA and MRSA colonised should undergo successful eradication therapy before training with buttonhole cannulation. In the group of patients in the maintenance phase, the use of an audit tool using a checklist is helpful to identify patients that are at high risk for access failure and intervention and retraining [26, 47–49]. As cannulation failure will not allow the patient to proceed with dialysis treatment, the patient should be instructed to abandon further attempts when appropriate, rest the access, apply ice for a minimum of 10 min and report to the training centre [50]. When possible, images of vascular access should be transferred and shared via a secured platform to assist in decision-making. If the problem cannot be readily resolved, the HHD training unit should review the patient the next day or perform a home visit.

The general vascular access alarms can be divided into venous and arterial pressure alarms. The arterial alarm may indicate line disconnection or access dysfunction, while venous alarm indicates thrombosis or clotting in the dialyzer or circuit, or kinking of lines. One of the most serious and life-threatening complications of HHD is venous needle dislodgement leading to significant blood loss, [32] and although this may not trigger the dialysis machine venous pressure alarm, wearing a wetness detector underneath the fistula alarm will trigger a separate alarm to alert the patients. As such wetness alarms are recommended for those HHD patients opting for nocturnal HHD. Training should highlight the importance of the alarms, the consequences of dismissing alarms, and troubleshooting steps. The use of educational tools such as the teach-back method should be considered to increase understanding and improve patient confidence [51, 52]. Competency checks much be performed during training and regularly in the maintenance phase. As some alarms may not often be triggered, we suggest a short and concise patient reminder card in addition to the HHD patient manual on troubleshooting these alarms as visual reminders [53, 54]. If the vascular access alarm is not resolved, patients are advised to contact HHD nursing or the technical hotline.

The recent Kidney Disease Outcomes Quality Initiative (KDOQI) Clinical Practice Guidelines for Vascular Access 2019 focused on regular physical examination and checking the vascular access to detect clinical indicators of flow dysfunction by trained and experienced dialysis nurses or physicians over regular access flow monitoring [50]. In the outpatient dialysis centre, vascular access surveillance is often conducted using various devices in addition to physical examination. However, this may be limited to purely clinical examination for HHD patients. Given that the KDOQI recommendations were based on weak evidence and the concern, in particular with reports of HHD patients at higher risk of vascular access thrombosis, possibly due to a higher intensity of dialysis, such as more prolonged and more frequent dialysis sessions, we suggest teaching patients to monitor and report access dysfunction and pseudo-aneurysm formation. This needs to be supplemented by physical examination by nursing and physician review during clinic encounters and six-monthly access flow monitoring [55, 56]. When required, the HHD patient will need to undergo retraining with an audit tool to ensure that they are able to detect, record, and report access problem promptly to the HHD nursing team [47]. The HHD program should collaborate closely with the vascular access team to facilitate urgent vascular access evaluation and intervention when needed [57].

## Discussion

The concept of the hospital at home (HAH) that substitutes hospital-level services at home for what would otherwise be inpatient hospitalisations has gained popularity over recent years, even locally, in Singapore [58]. Although HAH is distinctly different from HHD, which replaces an inpatient service rather than an outpatient service, the data reporting that HAH is safe and effective is promising to suggest the management of patients with acute medical illness in a home setting is safe [59–61]. Nevertheless, the data on safety is still relatively limited, and management in the home setting should be considered as supplementation to a healthcare facility rather than replacement in suitable individuals [62]. World-wide, there are different approaches to HHD, with some centers restricting access to programs for patients living alone, whereas others provide assistance at home, and even help with access cannulation and decannulation. As we were initiating a HHD program de-novo, we restricted patient selection to those with fistula access and a partner or carer at home.

While HHD shares some similarities with ICHD, it also varies significantly with a more selected group of patients that are relatively healthier and having lesser human interaction involved in their treatment. The similar prescription and processes in HHD allow implementation of a system in a more controlled environment. HHD can, therefore can be undertaken safely, if not safer despite performing a high complex treatment at home. Notably, the risk and the safety strategies for HHD vary from that of ICHD [63]. While the common safety problems at ICHD were falls, medication errors, access-related, dialyser prescription errors, and excess blood loss or prolonged bleeding, we identified vascular-access as the main safety area for HHD similar to that reported by Holey et al. [64, 65]. The risk in other areas was successfully reduced by the selection and detailed planning, and framework focussing on risk reduction.

Our safety framework for the HHD program is focused mainly on risk reduction and pre-emptive evaluation. This includes strict assessment criteria for the suitability, training with frequent competency checks with individualised treatment plans for patients where appropriate, and regular checks using a standardised audit tool [25, 66, 67]. Patient selection for HHD is a defined and vigorous process and includes a thorough clinical assessment, evaluation of the home environment as well as psychosocial background [68]. Subsequent to this assessment, the patient will need to undergo a period of training, which will vary between patients, initially aiming for for 8–16 weeks with multiple clinical and theoretical competency assessments at completion of each skill and weekly. For the first haemodialysis treatment at home, a dialysis nurse and technologist will be present to observe the patient performing the session correctly and monitor for any technical errors. If there are no problems and the patient is confident after the first session, the first clinic visit will then be scheduled 4 weeks later Thereafter, clinic visits are scheduled at monthly intervals for the initial 6 months. Patients are provided 24-h nursing and technical tele consult at all times [66]. Ad-hoc home visit arrangements during office hour can be made if needed or patients will be asked to return clinic for review by physician or additional test.Home visits are scheduled to alternate between clinic visits. We used FMEA in our study to pre-emptively identify processes that can fail, prioritise failure modes with the higher risk, and set risk reduction strategies.

In terms of HHD infrastructure and equipment, there are often two or more critical stakeholders involved, with one being the contractor responsible for plumbing modifications and electrical works, and the dialysis vendor(s) providing dialysis machines, portable reverse osmosis machine and other components to the dialysis water system. Depending upon the quality of the potable water, additional or larger water softeners, carbon filters may be required depending on of the local water supply. The contractor and installation company for HHD will need to be familiar with and ensure full compliance with all regulatory requirements Adequate assessment of space and placement of equipment with regular maintenance by the vendor will reduce the risk of failure. HHD machines designs and consumables such as blood lines should be chosen with considerations for simplicity, patient-friendly set-up and interface, easy to trouble shoot with features to improve safety [66, 69]. In the event of equipment failure, repairs will be initiated at the earliest possibility, and patients will be directed to dialyse at the HHD unit under the physician’s direction.

Self-cannulation competency is the most crucial competency in completing HHD training. Failure in cannulation will not allow dialysis to be initiated regardless of competency in other areas. While assisted home dialysis, both for HHD and PD is possible,there is a significant health care cost.. The manpower cost can be prohibitive, and there may also be a shortage of dialysis trained nurses. Even if the trained staff are only required to start and finish the treatment session, the required hours including travel time for the nurse adds a significant cost factor, especially in countries with high wages. Vascular access-related events were found to have the highest RPN in our assessment of the early transition and maintenance phase of HHD. This is consistent with previous reports that vascular access is the most common category leading to severe adverse events in the HHD programme, with calls received frequently deemed severe [8, 13, 70]. We discussed the main areas with the highest RPN as agreed by the two different teams. As many of the potential risk mitigation solutions overlap, we suggest a bundle approach to addressing vascular-access related failure (Table 4). As our program matures, we intend to include patients with catheter access in the programme but this will warrant a detailed review of risk and pre-emptive risk reduction strategies.

**Table 4:** Summary of recommendation for vascular access

Emphasis on vascular access in education and training
Specific focus on importance, consequences and how to troubleshoot for vascular access related incidents
Dedicated staff for self-cannulation training
Individualise cannulation therapy plan
Concise patient reminder cards
24 h access to dialysis nursing and vascular access hotline
Regular vascular access checks with audit tool [47]
Close collaboration with vascular access team
Retraining when necessary

Previously published data from well-established programmes reported no correlation between the experiences of HHD programme with the occurrence of adverse events but found that these events occurred primarily in patients with some degree of HHD experience with a median vintage of two years, suggesting that there may be some degree of experimentation, serendipity, complacency, burnout, and non-compliance [13, 47, 71]. Our new programme's framework will include a minimum of two monthly reviews either at the HHD training centre or at home with an audit as the patient is dialysing. We will emphasize specific attention every two years through a thorough review using an audit tool and theoretical competency assessment and sharing incidents in the programme to highlight the importance of adherence to protocols [47]. Similarly, a two-year review of the patient’s suitability to continue HHD will be conducted to ensure safety in continuing the treatment with progression or new cognitive and psychological changes. Experienced centres have reported technical issues and human error as a contributory cause to incidents in HHD programmes [13]. While our safety framework for the program is focused mainly on risk reduction and pre-emptive evaluation, not all failures can be wholly preventable, and measures will need to be put in place for the early detection of failure with an established workflow to resolve acute problems, with a rapid evaluation cycle to prevent future occurrences. Examples of early detection and workflow for acute access-related issues will include a wetness detector, blood sensors, or needle dislocation sensors both at the access site and under the fistula arm, coupled with accessibility to technical and nursing support [72, 73]. We will be conducting regular audit of the programme going forward and conducting concise incident analysis or root cause analysis adopting a latent approach with multidisciplinary team involvement, comprising physician, nursing, quality expert, technical team, and possibly patients with each near miss or incident reported [74, 75]. The action plan will be designed for prevention and early detection. Changes to workflow will be then subsequently made as necessary, emphasizing communication to all patients regarding any changes in a timely manner.

Failure mode and effects analysis (FMEA) is a proactive risk management tool for identifying the possible failure modes of a system, process, product or service, analysing the causes and effects of the failures, and eliminating or reducing the most significant ones by proposing risk mitigation actions [76]. FMEA is effective in evaluating both new and existing processes and systems. While FMEA has not been validated for HHD, we have used FMEA as an assessment tool before starting a new clinical program focussing on processes with highest risk. Given that there was no prior experience in HHD at our hospital, we sought collaboration from another unit. The involvement of the two HHD teams with different experiences and perspectives is unique and is also the strength of our study. It generates the opportunity to review HHD as a start-up with different challenges and to use the lessons from a mature HHD programme to help craft solutions to these challenges. The collaboration between the two teams provided an excellent platform for sharing and discussion as we focused on the safety aspects of our pilot HHD programme. The minor difference between HHD vascular access management between the two programmes are outlined in Table 5.

**Table 5:** Outline of differences between vascular access management in new and an established HHD programme

	New HHD programme	Established HHD programme
Frequency of review	Monthly for the first 3 months then, 2 monthly clinic review and physical examination of vascular access by HHD nurse and physician 3 monthly review of vascular access by vascular surgeon with vascular access scans if necessary	6 monthly clinic review and physical examination of vascular access by HHD nurse
Competency assessment	2 monthly review alternating home visit and HHD centre review by both HHD nurse and physician	6 monthly review alternating home visit and HHD centre review by HHD nurse

Although the processes of HHD are similar in many instances, the ability to generalise the failure modes and potential solutions for risk mitigation in two different settings may limit our study findings. There are inherent differences in our population in terms of logistics, demographics, and even simple differences such as climate and housing compared to that in London. To reduce unknown confounders related to these differences, we aim to conduct a second FMEA after starting our HHD programme and to examine our suggested solutions prospectively.

## Conclusions

Although HHD may appear to be a complex treatment performed by patients at home without direct supervision, HHD is a safe therapy as evident by its past record. Enhancing safety and patient experience will encourage and motivate patient to choose HHD [55]. The reported adverse incidents are similar to that of ICHD. However, HHD patients must have the ability to manage any treatment related complications occurring at home, occasionally with the presence of a caregiver and hence, the enhanced focus on safety and the reason for this FMEA approach. Our study reports the use of FMEA with the involvement of two teams, at different ends of the clinical experience scale in HHD. We summarize here the risk assessment of possible areas of failure in starting HHD from different perspectives. The risk reduction strategies are not new, but we have designed a framework that addresses the specific areas in a bundle approach. As vascular access related medical emergencies were the most prominent in our risk assessment, our FMEA was primarily directed to this area of risk and we successfully identified risk reduction strategies.

## Supplementary Information

**Additional file 1.** Possibleareas of failure during transitioning to home haemodialysis.

## Data Availability

All data generated during this study are included in this published article and supplementary information file.

## Abbreviations

HHD: Home haemodialysis
ICHD: In-centre (ICHD)
ESKF: End stage kidney failure
HD: Haemodialysis
FMEA: Failure mode effects analysis
NUH: National University Hospital Singapore
RFH: Royal Free Hospital
RPN: Risk priority number
RO: Reverse osmosis
MSSA: Methicillin-sensitive staphylococcus aureus
MRSA: Methicillin-resistant staphylococcus aureus
KDOQI: Kidney Disease Outcomes Quality Initiative
HAH: Hospital at home

## References

[1] Rydell H, Ivarsson K, Almquist M, Segelmark M, Clyne N (2019). Improved long-term survival with home hemodialysis compared with institutional hemodialysis and peritoneal dialysis: a matched cohort study. BMC Nephrol.

[2] Rydell H, Ivarsson K, Almquist M, Clyne N, Segelmark M (2019). Fewer hospitalizations and prolonged technique survival with home hemodialysis- a matched cohort study from the Swedish renal registry. BMC Nephrol.

[3] Masterson R (2008). The advantages and disadvantages of home hemodialysis. Hemodial Int.

[4] Watanabe Y, Ohno Y, Inoue T, Takane H, Okada H, Suzuki H (2014). Home hemodialysis and conventional in-center hemodialysis in Japan: a comparison of health-related quality of life. Hemodial Int.

[5] Pauly RP, Gill JS, Rose CL, Asad RA, Chery A, Pierratos A (2009). Survival among nocturnal home haemodialysis patients compared to kidney transplant recipients. Nephrol Dial Transplant.

[6] Nishio-Lucar AG, Bose S, Lyons G, Awuah KT, Ma JZ, Lockridge RS (2020). Intensive home hemodialysis survival comparable to deceased donor kidney transplantation. Kidney Int Rep.

[7] Sands JJ, Lacson E, Ofsthun NJ, Kay JC, Diaz-Buxo JA (2009). Home hemodialysis: a comparison of in-center and home hemodialysis therapy in a cohort of successful home hemodialysis patients. ASAIO J.

[8] Kraus M, Burkart J, Hegeman R, Solomon R, Coplon N, Moran J (2007). A comparison of center-based vs. home-based daily hemodialysis for patients with end-stage renal disease. Hemodial Int.

[9] Haroon S, Griva K, Davenport A (2020). Factors affecting uptake of home hemodialysis among self-care dialysis unit patients. Hemodial Int.

[10] Lee A, Gudex C, Povlsen JV, Bonnevie B, Nielsen CP (2008). Patients’ views regarding choice of dialysis modality. Nephrol Dial Transplant.

[11] Keating PT, Walsh M, Ribic CM, Brimble KS (2014). The impact of patient preference on dialysis modality and hemodialysis vascular access. BMC Nephrol.

[12] Office NROD (2018). Singapore renal registry annual report 2018.

[13] Wong B, Zimmerman D, Reintjes F, Courtney M, Klarenbach S, Dowling G (2014). Procedure-related serious adverse events among home hemodialysis patients: a quality assurance perspective. Am J Kidney Dis.

[14] Shebl NA, Franklin BD, Barber N (2009). Is failure mode and effect analysis reliable?. J Patient Saf.

[15] Bonfant G, Belfanti P, Paternoster G, Gabrielli D, Gaiter AM, Manes M (2010). Clinical risk analysis with failure mode and effect analysis (FMEA) model in a dialysis unit. J Nephrol.

[16] Martin LD, Grigg EB, Verma S, Latham GJ, Rampersad SE, Martin LD (2017). Outcomes of a failure mode and effects analysis for medication errors in pediatric anesthesia. Paediatr Anaesth.

[17] Potts HW, Anderson JE, Colligan L, Leach P, Davis S, Berman J (2014). Assessing the validity of prospective hazard analysis methods: a comparison of two techniques. BMC Health Serv Res.

[18] Joint Commission Resources Inc. Failure Mode and Effects Analysis in Health Care: Proactive Risk Reduction. 3rd edition. Joint Commission Resources, Oakbrook Terrace, IL; 2010.

[19] Ashley L, Armitage G, Neary M, Hollingsworth G (2010). A practical guide to failure mode and effects analysis in health care: making the most of the team and its meetings. Jt Comm J Qual Patient Saf.

[20] Complied by Department of Defense Patient Safety Center - AFIP for the AF Patient Safety Program. Failure Mode and Effects Analysis (FMEA): An Advisor’s Guide.; Version 1.0 June 2004. https://docplayer.net/16235308-An-advisor-s-guide-version-1-0-june-2004.html.

[21] Ho CC, Liao CJ (2011). The use of failure mode and effects analysis to construct an effective disposal and prevention mechanism for infectious hospital waste. Waste Manag.

[22] Li X, He M, Wang H (2017). Application of failure mode and effect analysis in managing catheter-related blood stream infection in intensive care unit. Medicine (Baltimore).

[23] Labriola L, Crott R, Desmet C, Andre G, Jadoul M (2011). Infectious complications following conversion to buttonhole cannulation of native arteriovenous fistulas: a quality improvement report. Am J Kidney Dis.

[24] Kaplowitz LG, Comstock JA, Landwehr DM, Dalton HP, Mayhall CG (1988). Prospective study of microbial colonization of the nose and skin and infection of the vascular access site in hemodialysis patients. J Clin Microbiol.

[25] Nesrallah GE, Mustafa RA, MacRae J, Pauly RP, Perkins DN, Gangji A (2013). Canadian Society of Nephrology guidelines for the management of patients with ESRD treated with intensive hemodialysis. Am J Kidney Dis.

[26] Faratro R, Jeffries J, Nesrallah GE, MacRae JM (2015). The care and keeping of vascular access for home hemodialysis patients. Hemodial Int.

[27] Vascular Access Special Interest Group BRS (2016). Clinical practice recommendations for use of buttonhole technique for cannulation of arteriovenous fistulae.

[28] Kang JS, Jang HR, Lee JE, Park YJ, Rhee H, Seong EY (2016). The bacterial colonization in tunneled cuffed dialysis catheter and its effects on residual renal function in incident hemodialysis patients. Clin Exp Nephrol.

[29] Kang YC, Tai WC, Yu CC, Kang JH, Huang YC (2012). Methicillin-resistant Staphylococcus aureus nasal carriage among patients receiving hemodialysis in Taiwan: prevalence rate, molecular characterization and de-colonization. BMC Infect Dis.

[30] Narasimha Krishna V, Allon M (2015). What is the significance of Staphylococcus aureus colonization in hemodialysis patients?. Nephron.

[31] Nesrallah GE, Cuerden M, Wong JH, Pierratos A (2010). Staphylococcus aureus bacteremia and buttonhole cannulation: long-term safety and efficacy of mupirocin prophylaxis. Clin J Am Soc Nephrol.

[32] Van Waeleghem JP, Chamney M, Lindley EJ, Pancirova J (2008). Venous needle dislodgement: how to minimise the risks. J Ren Care.

[33] Chan DYF, Dobson S, Barber T (2021). Hemodialysis taping styles and their effect on reducing the chance of venous needle dislodgement. Semin Dial.

[34] Implementing Hemodiaysis in the home ISHD. 2016. https://www.ishd.org/library/pdfs/Module_7_Apdx_TapingIVNeedle.pdf. Accessed 31 November 2022.

[35] Hodde L, Sandroni S (1992). Emergency department evaluation and management of dialysis patient complications. J Emerg Med.

[36] Larsen C, Weathers B, Schwartzwald M, Barton M. Focus on: dialysis access emergencies. American College of Emergency Physicians Clinical & Practice Management 2010. https://www.acep.org/Clinical—Practice-Management/Focus-On-Dialysis-Access-Emergencies/. Accessed 31 November 2022.

[37] Davenport A (2011). What are the anticoagulation options for intermittent hemodialysis?. Nat Rev Nephrol.

[38] Karkar A, Hegbrant J, Strippoli GF (2015). Benefits and implementation of home hemodialysis: a narrative review. Saudi J Kidney Dis Transpl.

[39] Mott S, Moore H (2009). Using 'Tandem hand' technique to facilitate self-cannulation in hemodialysis. Nephrol Nurs J..

[40] Verhallen AM, Kooistra MP, van Jaarsveld BC (2007). Cannulating in haemodialysis: rope-ladder or buttonhole technique?. Nephrol Dial Transplant.

[41] Mott S, Schatell D, Patterson P, Last FM (2020). Techniques for self-cannulation. Nephrol Nurs J.

[42] Staaf K, Fernstrom A, Uhlin F (2021). Cannulation technique and complications in arteriovenous fistulas: a Swedish renal registry-based cohort study. BMC Nephrol.

[43] Casey JR, Hanson CS, Winkelmayer WC, Craig JC, Palmer S, Strippoli GF (2014). Patients' perspectives on hemodialysis vascular access: a systematic review of qualitative studies. Am J Kidney Dis.

[44] Wong B, Muneer M, Wiebe N, Storie D, Shurraw S, Pannu N (2014). Buttonhole versus rope-ladder cannulation of arteriovenous fistulas for hemodialysis: a systematic review. Am J Kidney Dis.

[45] Lyman M, Nguyen DB, Shugart A, Gruhler H, Lines C, Patel PR (2020). Risk of vascular access infection associated with buttonhole cannulation of fistulas: data from the national healthcare safety network. Am J Kidney Dis.

[46] Grudzinski A, Mendelssohn D, Pierratos A, Nesrallah G (2013). A systematic review of buttonhole cannulation practices and outcomes. Semin Dial.

[47] Rousseau-Gagnon M, Faratro R, D'Gama C, Fung S, Wong E, Chan CT (2016). The use of vascular access audit and infections in home hemodialysis. Hemodial Int.

[48] Audit tool for AV Fistula/ Graft Cannulation. 2017. https://www.cdc.gov/dialysis/PDFs/collaborative/AV-Fistula-Graft-Cannulation-Observations.pdf. Accessed 31 November 2022.

[49] Audit tool for AV Fistula/ Graft Decannulation. 2017. https://www.cdc.gov/dialysis/PDFs/collaborative/AV-Fistula-Graft-Decannulation-Observations.pdf. Accessed 31 November 2022.

[50] Lok CE, Huber TS, Lee T, Shenoy S, Yevzlin AS, Abreo K (2020). KDOQI Clinical Practice Guideline for Vascular Access: 2019 Update. Am J Kidney Dis.

[51] Ryan-Madonna M, Levin RF, Lauder B (2019). Effectiveness of the teach-back method for improving caregivers’ confidence in caring for hospice patients and decreasing hospitalizations. J Hosp Palliat Nurs.

[52] Yen PH, Leasure AR (2019). Use and effectiveness of the teach-back method in patient education and health outcomes. Fed Pract.

[53] Chae SY, Chae MH, Isaacson N, James TS (2009). The patient medication list: can we get patients more involved in their medical care?. J Am Board Fam Med.

[54] Lachowsky M, Levy-Toledano R (2002). Improving compliance in oral contraception: 'the reminder card'. Eur J Contracept Reprod Health Care.

[55] Rocco MV, Lockridge RS, Beck GJ, Eggers PW, Gassman JJ, Greene T (2011). The effects of frequent nocturnal home hemodialysis: the frequent hemodialysis network nocturnal trial. Kidney Int.

[56] Chertow GM, Levin NW, Beck GJ, Depner TA, Eggers PW, Group FHNT (2010). In-center hemodialysis six times per week versus three times per week. N Engl J Med.

[57] Schmidli J, Widmer MK, Basile C, de Donato G, Gallieni M, Gibbons CP (2018). Editor’s choice - vascular access: 2018 clinical practice guidelines of the European Society for Vascular Surgery (ESVS). Eur J Vasc Endovasc Surg.

[58] Lai YF, Lim YW, Kuan WS, Goh J, Soong JTY, Shorey S, Ko SQ (2021). Asian attitudes and perceptions toward hospital-at-home: a cross-sectional study. Front Public Health.

[59] ‘Hospital at home’ approach is safe and effective. 2016. https://www.nursingtimes.net/news/community-news/hospital-at-home-approach-is-safe-and-effective-29-06-2016/. Accessed 31 November 2022.

[60] Rickert J (2022). On patient safety: hospital-at-home care seems like a winner, but is it safe for our patients?. Clin Orthop Relat Res.

[61] Montalto M (1998). How safe is hospital-in-the-home care?. Med J Aust.

[62] Foley OW, Ferris TG, Thompson RW, Heng M, Ricciardi R, Del Carmen MG, Safavi KC (2021). Potential impact of hospital at home on postoperative readmissions. Am J Manag Care.

[63] Arenas Jimenez MD, Ferre G, Alvarez-Ude F (2017). Strategies to increase patient safety in hemodialysis: application of the modal analysis system of errors and effects (FEMA system). Nefrologia.

[64] Holley JL (2006). A descriptive report of errors and adverse events in chronic hemodialysis units. Nephrol News Issues.

[65] Garrick R, Kliger A, Stefanchik B (2012). Patient and facility safety in hemodialysis: opportunities and strategies to develop a culture of safety. Clin J Am Soc Nephrol.

[66] Rajkomar A, Farrington K, Mayer A, Walker D, Blandford A (2014). Patients' and carers' experiences of interacting with home haemodialysis technology: implications for quality and safety. BMC Nephrol.

[67] Agarwal AK, Boubes KY, Haddad NF (2021). Essentials of vascular access for home hemodialysis. Adv Chronic Kidney Dis.

[68] Rioux JP, Marshall MR, Faratro R, Hakim R, Simmonds R, Chan CT (2015). Patient selection and training for home hemodialysis. Hemodial Int.

[69] Haroon S, Davenport A (2018). Haemodialysis at home: review of current dialysis machines. Expert Rev Med Devices.

[70] Reintjes F, Herian N, Shah N, Pauly RP (2019). Prospective monitoring of after-hours nursing and technologist support calls to a regional Canadian home hemodialysis program. Hemodial Int.

[71] Hawley CM, Jeffries J, Nearhos J, Van Eps C (2008). Complications of home hemodialysis. Hemodial Int.

[72] Polaschegg HD (2010). Venous needle dislodgement: the pitfalls of venous pressure measurement and possible alternatives, a review. J Ren Care.

[73] Ahlmen J, Gydell KH, Hadimeri H, Hernandez I, Rogland B, Strombom U (2008). A new safety device for hemodialysis. Hemodial Int.

[74] Leape LL (1997). A systems analysis approach to medical error. J Eval Clin Pract.

[75] Using Checklists and Audit Tools. 2015. https://www.ahrq.gov/patient-safety/settings/esrd/resource/checklist.html#:~:text=Resources-,Using%20Checklists%20and%20Audit%20Tools,-ESRD%20Toolkit. Accessed 31 November 2022.

[76] Stamatis DH (2023). Failure Mode and Effect Analysis: FMEA from Theory to Execution.

