# Supplementary material for: Risk assessment of failure during transitioning from in-centre to home haemodialysis
Source: BMC Nephrol. 2022 Dec 20;23:406. doi: 10.1186/s12882-022-03039-4 (PMC9768953; doi:10.1186/s12882-022-03039-4)
Supplement: Supplementary file 1 — Additional file 1. Possibleareas of failure during transitioning to home haemodialysis. [file 12882_2022_3039_MOESM1_ESM.docx]

**Possible areas of failure during transitioning to home haemodialysis**

| **Processes** | **Failure mode** | **Effect/ Consequences** | **Likelihood** | **Severity** | **Detectability** | **RPN** |
| --- | --- | --- | --- | --- | --- | --- |
| **1** | **Setting up HHD system at home** |  |  |  |  |  |
| Setting up HD Machine | Machine cannot be placed in the space at home | Not suitable for HHD | 1 | 4 | 1 | 4 |
| Setting up water treatment system | Portable RO cannot be placed in the space at home | Not suitable for HHD | 1 | 4 | 1 | 4 |
|  | Connection of portable RO and machine cannot be achieved | Unable to perform HHD | 1 | 4 | 1 | 4 |
| Setting up drainage | Violating municipal standards for discharge of dialysis effluent | Not suitable for HHD | 1 | 4 | 1 | 4 |
|  | Incorrect technical requirement for height of drainage hole | Unable to perform HHD | 1 | 4 | 1 | 4 |
| Setting up and establishing power and water supply | Power socket not suitable for machine / portable RO | Not suitable for HHD | 1 | 4 | 1 | 4 |
|  | Power socket not sited correctly | Unable to perform HHD | 1 | 4 | 1 | 4 |
|  | Connector to water point incompatible | Unable to perform HHD | 1 | 4 | 1 | 4 |
|  | Water points not sited correctly | Unable to perform HHD | 1 | 4 | 1 | 4 |
|  | Inadequate water pressure to operate the portable RO | Unable to perform HHD | 1 | 4 | 1 | 4 |
|  | Water temperature too high or too low | Unable to perform HHD | 1 | 4 | 1 | 4 |
| Creating storage and getting consumables ready | Lack of storage for consumables | Not suitable for HHD | 1 | 4 | 1 | 4 |
|  | Consumables passed shelf-life (beyond expiration date) | Unable to perform HHD | 1 | 4 | 1 | 4 |
| **2** | **Completing test prior to starting HHD** | | | | | |
| Checking water treatment system | Failure to achieve meet minimum safety and quality levels of dialysis water and fluid requirement | Haemolysis, Sepsis  Unable to perform HHD | 3 | 4 | 1 | 12 |
| Initiating HD machine self-test | Repeatedly fails self-test | Unable to perform HHD | 1 | 4 | 1 | 4 |
| **3** | **Performing HHD** | | | | | |
| **3A** | **Preparation to start HHD treatment** | | | | | |
| Starting HD Machine | Machine cannot be switched on | Unable to perform HHD | 1 | 4 | 1 | 4 |
|  | Machine breakdown | Unable to perform HHD | 1 | 4 | 1 | 4 |
| Starting water treatment system | Portable reverse osmosis (RO) cannot be switched on | Unable to perform HHD | 1 | 4 | 1 | 4 |
|  | Portable RO breakdown | Unable to perform HHD | 1 | 4 | 1 | 4 |
| Ensuring drainage | Inadequate water or dialysate flow | Unable to perform HHD | 1 | 4 | 1 | 4 |
|  | Inadequate water pressure | Unable to perform HHD | 1 | 4 | 1 | 4 |
|  | Blocked drainage | Unable to perform HHD | 1 | 4 | 1 | 4 |
|  | Flooding from cracked lines or choked drainage | Unable to perform HHD | 1 | 4 | 1 | 4 |
| Starting power and water supply | Interruption in water supply | Unable to perform HHD | 1 | 4 | 1 | 4 |
|  | Interruption in power supply | Unable to perform HHD | 1 | 4 | 1 | 4 |
| Gathering dialysis consumables | No or insufficient supply of consumables needed for treatment | Unable to perform HHD | 1 | 4 | 1 | 4 |
|  | Incorrect supply of consumables | Unable to perform HHD | 1 | 4 | 1 | 4 |
|  | Failure to supply heparin | Clotting of extracorporeal circuit and interruption to dialysis | 1 | 6 | 1 | 6 |
|  | Failure to supply disinfectant | Unable to disinfect machine after dialysis treatment | 1 | 4 | 1 | 4 |
| **3B** | **General patient and dialysis equipment evaluation before starting dialysis** | | | | | |
| General evaluation | Starting dialysis when unwell | Loss of consciousness, Fever, Hypotension, Chest pain | 3 | 10 | 3 | 90 |
| Priming and connectivity of dialysis blood lines | Poor connection | Spillage of blood on the floor / acute blood loss  Unable to proceed priming  Tugging of A blood line / High V pressure  Clotting of extra-corporeal circuit | 3 | 8 | 3 | 72 |
|  | Incorrect connection | Spillage of blood on the floor / acute blood loss  Unable to proceed priming | 3 | 8 | 3 | 72 |
|  | Kinked blood lines | Tugging of A blood line / High V pressure  Clotting of extra-corporeal circuit | 3 | 8 | 3 | 72 |
| Measuring weight | Error in weight taken | Hypotension, cramps, Chest Pain, Arrhythmias, Nausea | 2 | 6 | 4 | 48 |
|  | Incorrect dry weight | Hypotension, cramps, Chest Pain, Arrhythmias | 2 | 6 | 4 | 48 |
| Deciding and calculation of ultrafiltration | Excessive ultrafiltration | Hypotension, cramps, Chest Pain, Arrhythmias | 2 | 6 | 5 | 60 |
|  | Inadequate ultrafiltration | Hypertension, Shortness of breath  Unable to attain dry weight | 2 | 8 | 4 | 64 |
| Taking medications before dialysis | Taking excessive antihypertensive medications | Hypotension | 3 | 8 | 4 | 96 |
|  | Forgot to take antihypertensive or taking lower dose | Hypertension | 3 | 8 | 4 | 96 |
| **3C** | **Managing vascular access during dialysis treatment** | | | | | |
| Cleaning of access site | Non-compliance to cleaning of access site | Vascular access infection and septicaemia  Unable to proceed with haemodialysis using vascular access | 2 | 8 | 6 | 96 |
| Scab removal for those of buttonhole cannulation | Incomplete scab removal for buttonhole cannulation | Vascular access infection and septicaemia  Unable to proceed with haemodialysis using vascular access | 2 | 9 | 8 | 144 |
| Establishing access cannulation | Unsuccessful access cannulation after three attempts | Unable to proceed with haemodialysis | 3 | 3 | 1 | 9 |
| Cannulation technique | Defective technique in cannulation access | Acute blood loss from venous extravasation and hematoma  Vascular access infection and septicaemia  Unable to proceed with haemodialysis using vascular access | 4 | 9 | 7 | 252 |
| Securing vascular access | Poor fixation of needles to skin, traction of circuit line or movement especially during nocturnal dialysis | Anaemia symptoms  Acute blood loss | 3 | 10 | 5 | 150 |
| Troubleshooting alarm related to vascular access | Failure to respond to arterial and venous pressure alarm | Acute blood loss  Hypotensive shock and death if excessive blood loss | 3 | 10 | 6 | 180 |
| Monitoring vascular access during dialysis | Failure to monitor vascular access during dialysis | Vascular access thrombosis  Bleeding from needling sites  Vascular access rupture  Acute blood loss causing hypotensive shock and death if excessive blood loss  Unable to proceed with haemodialysis using vascular access | 4 | 8 | 8 | 256 |
| Vascular access needles removal | Excessive and prolonged bleeding after removal of dialysis needles | Anaemia symptoms  Acute blood loss | 2 | 5 | 5 | 50 |
| Monitoring vascular access (general) | Failure to identify access related infection | Unable to proceed with haemodialysis using vascular access | 2 | 8 | 5 | 80 |
| **3D** | **Dealing with treatment interruption and troubleshooting machine alarms** | | | | | |
| Reprogramming after temporary interruption | Failure to reprogram after disconnection | Hypotension, cramps, Tachycardia | 3 | 8 | 4 | 96 |
| Troubleshooting dialysis machine alarms | Dialysate (conductivity and temperature) alarm trigger | Unable to continue dialysis treatment | 1 | 5 | 1 | 5 |
|  | Air detection alarm trigger | Unable to continue dialysis treatment | 1 | 10 | 1 | 10 |
|  | Blood leak alarm trigger | Unable to continue dialysis treatment | 1 | 5 | 1 | 5 |
| Calling for help | Unable to reach nursing or technical assistant for advice | Unable to continue dialysis treatment | 1 | 5 | 1 | 5 |
| Emergency during dialysis treatment | Need for emergency evacuation | Unable to continue dialysis treatment | 1 | 10 | 1 | 10 |
| **3E** | **Administering medications on dialysis** | | | | | |
| Administering anticoagulation | Excessive heparin administered | Excessive bleeding post dialysis | 2 | 4 | 2 | 16 |
| Administering new medications or using new consumables | Allergic reaction | Shortness of breath, Acute itch, Rash, Hypotension, Tachycardia | 2 | 8 | 1 | 16 |
| **3F** | **Others** | | | | | |
| Caregiver assisting HHD | Needle-stick injury to family member or caregiver | Risk of transmission of infection | 1 | 10 | 1 | 10 |
| **3G** | **Ending dialysis** | | | | | |
| Disposal of HD items | Improper of disposal biohazard waste | Unable to dispose waste  Risk of transmission of infection | 1 | 2 | 1 | 2 |
|  | Sharps box missing | Unable to dispose sharps | 1 | 5 | 1 | 5 |
